# The Conformational Contribution to Molecular Complexity and Its Implications for Information Processing in Living Beings and Chemical Artificial Intelligence

**DOI:** 10.3390/biomimetics9020121

**Published:** 2024-02-19

**Authors:** Pier Luigi Gentili

**Affiliations:** Department of Chemistry, Biology, and Biotechnology, Università degli Studi di Perugia, 06123 Perugia, Italy; pierluigi.gentili@unipg.it; Tel.: +39-0755855576

**Keywords:** chemistry, conformational stereoisomers, entropy, proteins, antibodies, intrinsically disordered proteins, DNA, RNA, fuzzy logic, chemical robotics

## Abstract

This work highlights the relevant contribution of conformational stereoisomers to the complexity and functions of any molecular compound. Conformers have the same molecular and structural formulas but different orientations of the atoms in the three-dimensional space. Moving from one conformer to another is possible without breaking covalent bonds. The interconversion is usually feasible through the thermal energy available in ordinary conditions. The behavior of most biopolymers, such as enzymes, antibodies, RNA, and DNA, is understandable if we consider that each exists as an ensemble of conformers. Each conformational collection confers multi-functionality and adaptability to the single biopolymers. The conformational distribution of any biopolymer has the features of a fuzzy set. Hence, every compound that exists as an ensemble of conformers allows the molecular implementation of a fuzzy set. Since proteins, DNA, and RNA work as fuzzy sets, it is fair to say that life’s logic is fuzzy. The power of processing fuzzy logic makes living beings capable of swift decisions in environments dominated by uncertainty and vagueness. These performances can be implemented in chemical robots, which are confined molecular assemblies mimicking unicellular organisms: they are supposed to help humans “colonise” the molecular world to defeat diseases in living beings and fight pollution in the environment.

## 1. Introduction

Molecular Complexity is a highly debated topic that interests many fields, such as synthetic organic chemistry [1,2,3,4] and drug discovery [5,6,7,8], the origin of life and biology [9,10,11], astrochemistry [12,13], Chemical Artificial Intelligence [14,15,16], and conformational statistics [17,18,19]. Unfortunately, a unique and universally accepted definition of Molecular Complexity does not exist. Indeed, it is fair to state that it refers to the structures of the molecules, and it is well-established that the molecular structures rule the behavior of molecules.

Several algorithms have been proposed to quantify Molecular Complexity [20]. Most of these algorithms are based on representing molecules as two-dimensional graphs and use Shannon’s entropy formula (see Equation (1)) [21]. Any molecular structure constitutes a message carrying a certain amount of information or entropy, the more significant the amount of this information, the larger the Complexity of the structure.

(1) $$H\left(X\right)=-\sum_{i}p(x_{i})\cdot \log_{2}p(x_{i})$$

In Equation (1), $p(x_{i})$ represents the frequency of a particular structural feature ($x_{i}$) such as single atoms (for instance, C, N, O, and so on) or atomic groups (for instance, the carbonyl C=O or the imine C=N groups). When a molecule is described through the laws of quantum physics, each electron will be represented by a wavefunction $\psi_{j}(\vec{r})$, and a density $\rho_{j}\left(\vec{r}\right)={\left|\psi_{j}(\vec{r})\right|}^{2}$. The value of the density $\rho_{j}\left(\vec{r}\right)$ is proportional to the probability of finding the *j*-th electron in a particular point, $\vec{r}$, of the three-dimensional space. If $\rho (\vec{r})$ represents the density of all the electrons, the integral $\int \rho (\vec{r})d\vec{r}$ represents the total number of electrons (N). If $\bar{\rho }\left(\vec{r}\right)=\rho (\vec{r})/N$ is the molecular electron density normalized to unity (i.e., the so-called shape function) [22], it is a probability of finding electrons in the different spatial points. Therefore, it can be used in the definition of Shannon’s Entropy:

(2) $$H\left(X\right)=-\int \bar{\rho }\left(\vec{r}\right)\cdot log(\bar{\rho }\left(\vec{r}\right))\cdot d\vec{r}$$

Other algorithms exploit simple topological or physicochemical descriptors (for instance, the number of chiral centers, the molecular weight value, the number of rings, the number of topological torsions, and so on) to determine Molecular Complexity [1,2,4,23,24]. However, they need parametrization that can only be performed within specific classes of molecules.

Recently, two new approaches, which are based on molecular fragmentation, have been proposed. One is purely theoretical and involves fractal geometry [25]. A fractal dimension, d, of any molecule is calculated through the equation below:

(3) $$d=\underset{1/\gamma \to 0}{\lim}\left(\frac{log(N)}{log(\gamma )}\right)$$

In Equation (3), γ is the number of bonds and N is the number of distinct subgraphs obtained as fragments of the original graph. The fractal dimension becomes a quantitative estimation of Molecular Complexity in analogy to other research fields [26,27]. The other approach evaluates the minimum number of steps to reconstruct a molecule, starting from its irreducible parts made of bonds and atoms [11]. This latter approach is supported by experimental data collected by tandem mass spectrometry.

As far as we know, in all the approaches and algorithms proposed so far for estimating Molecular Complexity, no one considers the contribution of the conformations, although they often play relevant roles in chemical reactivity. This work aims to highlight and quantitatively characterize the contribution of conformations and has the following structure:

Paragraph 2 presents the types of information required to specify the structure of a molecule.

Paragraph 3 introduces Conformational Entropy.

Paragraph 4 explains that any conformational distribution works as a fuzzy set, and introduces Fuzzy Entropy.

Paragraph 5 reports some examples of the relevant roles played by conformations in biopolymers.

Paragraph 6 highlights that the logic of life is intrinsically fuzzy.

Paragraph 7 proposes a promising strategy to implement fuzzy logic in Chemical Artificial Intelligence and Robotics.

## 2. Hierarchical Description of a Molecular Structure

It is well-known that the behavior of any molecular compound depends on its structure. The definition of a molecular structure requires three types of information.

**Figure 1:**
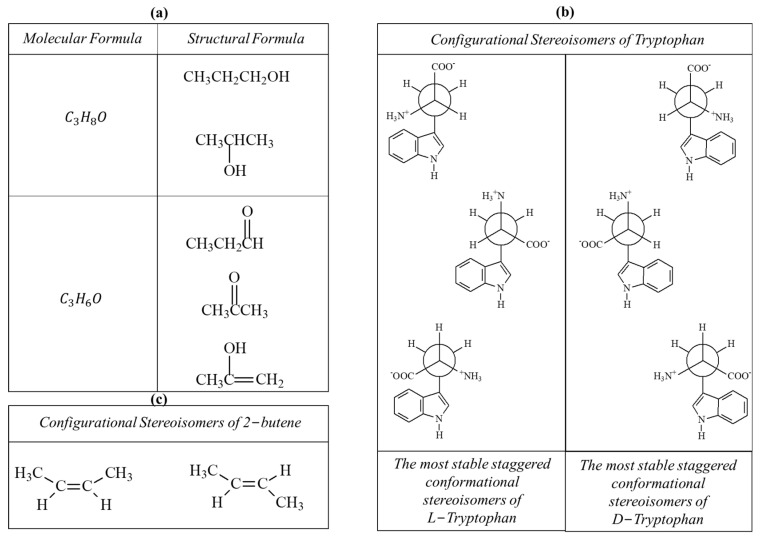
Examples of Structural Isomers that have the same molecular formulas but distinct structural formulas in (**a**). Difference between configurational and conformational isomers of Tryptophan in (**b**). Examples of other configurational stereoisomers in (**c**).

Firstly, the “Molecular Formula” (see Figure 1a) specifies the types and numbers of each chemical element within the molecule. The Molecular Formula includes information regarding the types and number of subatomic particles (electrons, protons, and neutrons) associated with every chemical element if the isotopes for each element are explicitly indicated.

Then, it is required to describe the order and how the atoms are bound. This information is included in the “Structural Formula” (see Figure 1a) that defines the molecular skeleton. Molecules with the same Molecular Formulas but different Structural Formulas are called structural isomers. If each molecule is viewed as a network or a graph [28], having the atoms as nodes and the covalent bonds as edges, the information contained in its Structural Formula can be translated into a peculiar adjacency matrix ${\bar{A}}_{SF}$ (see Equation (4)). The rank of the matrix equals the number of the atoms $N_{A}$, and each entry of the matrix, $a_{i,j}$, will be 0 if the atoms i and j are not bound, and it will be different from 0 otherwise.

(4) $${\bar{A}}_{SF}=\left(\begin{array}{ccc} a_{1,1} & \cdots & a_{1,N_{A}}\\ \vdots & a_{i,j} & \vdots \\ \vdots & \ddots & \vdots \\ a_{N_{A},1} & \cdots & a_{N_{A},N_{A}} \end{array}\right)$$

${\bar{A}}_{SF}$ will be particularly informative if every term $a_{i,j}\neq 0$ reports the absolute strength of the covalent bond linking the pair of i and j atoms. Alternatively, it might be the bond’s length.

Finally, it is essential to know the relative orientation of the atoms in the three-dimensional (3-D) space. Atoms and atomic groups that are not covalently bound but are spatially close, will interplay through electrostatic forces (such as the dipole-dipole and dipole-quadrupole interactions). The new adjacency matrix ${\bar{G}}_{3D}$ (see Equation (5)) will have two types of non-null terms, $g_{i,j}\neq 0$: one type relative to covalent bonds and already included in ${\bar{A}}_{SF}$, and another type relative to the electrostatic interactions established through the 3D space. Each $g_{i,j}$ can be the bond’s strength or the bond’s distance.

(5) $${\bar{G}}_{3D}=\left(\begin{array}{ccc} g_{1,1} & \cdots & g_{1,N_{A}}\\ \vdots & g_{i,j} & \vdots \\ \vdots & \ddots & \vdots \\ g_{N_{A},1} & \cdots & g_{N_{A},N_{A}} \end{array}\right)$$

Molecules with identical Molecular and Structural Formula (and hence identical adjacency matrices ${\bar{A}}_{SF}$), but different orientations of the atoms in 3-D space (and hence distinct adjacency matrices ${\bar{G}}_{3D})$ are called stereoisomers. Stereoisomers can be either configurational or conformational. They are configurational if the transformation from one stereoisomer to the other requires the breakage of at least one covalent bond, hence a few eV. They are conformational if the transition from one stereoisomer to another does not require the breakage of any covalent bond. Instead, it is required to overcome energetic barriers that are usually as high as or ten times higher than the thermal energy available at room temperature (i.e., kT≈0.02 eV). Figure 1b shows that the chiral amino-acid Tryptophan exists as two configurational stereoisomers: L- and D-Tryptophan. They are specular but not overlapping structures and are called enantiomers. Both L- and D-Tryptophan exist as two collections of conformational isomers. Three staggered conformers for both enantiomers are depicted in Figure 1b. These staggered structures are among the most stable conformers because the steric hindrances are minimized (i.e., the repulsive electrostatic interactions between the negative electronic charges are minimized). The transformation rate of one conformer to another depends on the energetic barrier height that must be overcome: the higher the barrier, the slower the transformation rate. At the limit, when these barriers are as high as the thermal energy available, the transformation is ultrafast. It occurs in the time scale of single vibrations (i.e., $10^{-13}s$). Intramolecular steric hindrances due to bulky molecular groups, intramolecular hydrogen bonds, or viscous micro-environments might hinder and reduce conformational dynamicity, which refers to the degree of freedom to move from one conformer to another using the available thermal energy. Also, low temperatures slow down the conformational dynamicity.

Finally, Figure 1c reports another example of configurational stereoisomers: the *cis*- and *trans*-2-Butene. They are called geometrical isomers, and it is possible to transform one stereoisomer into the other only after breaking the π bond of the C=C group.

Any compound that exists as a collection of $N_{C}$ conformational stereoisomers must be represented by an ensemble $\bar{\Gamma }$ of $N_{C}$ adjacency matrices of the type ${\left({\bar{G}}_{3D}\right)}_{k}$, with $k=1,2,\ldots ,N_{C}$, each multiplied by a weight coefficient $w_{k}$, which represents the relative abundance of the k-th conformer:

(6) $$\bar{\Gamma }=({w_{1}\left({\bar{G}}_{3D}\right)}_{1},w_{2}{\left({\bar{G}}_{3D}\right)}_{2},\ldots {w_{k}\left({\bar{G}}_{3D}\right)}_{k},\ldots ,{w_{N_{C}}\left({\bar{G}}_{3D}\right)}_{N_{C}})$$

with

(7) $${\left({\bar{G}}_{3D}\right)}_{k}=\left(\begin{array}{ccc} {\left(g_{1,1}\right)}_{k} & \cdots & {\left(g_{1,N_{A}}\right)}_{k}\\ \vdots & {\left(g_{i,j}\right)}_{k} & \vdots \\ \vdots & \ddots & \vdots \\ {\left(g_{N_{A},1}\right)}_{k} & \cdots & {\left(g_{N_{A},N_{A}}\right)}_{k} \end{array}\right)$$

The sum of all the weight coefficients $w_{k}$ is equal to 1:

(8) $$\sum_{k=1}^{N_{C}}w_{k}=1$$

The physicochemical properties of any compound, and particularly its chemical reactivity, depend on $\bar{\Gamma }$, i.e., its conformational distribution. Furthermore, $\bar{\Gamma }$ is context-dependent. In other words, the conformational distribution of any compound is strongly affected by the physicochemical features of the surrounding micro-environment. The physicochemical context (pcc) is usually described by specifying the values of macroscopic parameters such as temperature (T), pressure (P), volume (V), surface area extension (A), and chemical composition (C):

(9) pcc=f(T,P,V,A,C)

The chemical composition C affects other relevant features of the medium, such as its polarity and viscosity. pcc is a multivariable vector-valued function. If the variables T,P and C (appearing in Equation (9)) assumes just one uniform value in space, the micro-environment is homogeneous. In this case, there is just one context and hence just one $\bar{\Gamma }$ (see Equation (6)). On the other hand, when at least one of the three parameters T,P and C are not uniform in space, the micro-environment is heterogeneous. In this case, there will be more than one context, i.e., ${pcc}_{1},{pcc}_{2},\ldots$, and hence more than one $\bar{\Gamma }$: ${\left(\bar{\Gamma }\right)}_{{pcc}_{1}}$, ${\left(\bar{\Gamma }\right)}_{{pcc}_{2}}$, and so on, with as many $\bar{\Gamma }$ as are the number of distinct contexts. It is worthwhile noticing that the conformational distribution might depend on how a physicochemical context is reached, i.e., the path and how fast the path is traced.

## 3. Conformational Entropy

The knowledge of $\bar{\Gamma }$ (see Equation (6)) requires the determination of the $N_{C}$ adjacency matrices ${\left({\bar{G}}_{3D}\right)}_{k}$, and their relative weights $w_{k}$, with $k=1,\;2,\ldots ,N_{C}.$ Such determination can be accomplished either computationally or experimentally.

Computationally, the structures of the most stable conformers for a particular compound can be determined by solving the Schrödinger equations. If the $N_{C}$ conformers’ collection is at the thermodynamic equilibrium and abides by the Maxwell and Boltzmann statistics, and $E_{k}$ is the potential energy of the *k*-th conformer, the probability of the *k*-th conformer will be:

(10) $$p_{k}=\frac{e^{-\left(\frac{E_{k}}{kT}\right)}}{\sum_{k=1}^{N_{C}}e^{-\left(\frac{E_{k}}{kT}\right)}}$$

Based on the quantum-mechanical simulations and Equation (10), it is possible to define Conformational Entropy using Information (Shannon) Entropy [21]:

(11) $$S_{CC}=-\sum_{k=1}^{N_{C}}p_{k}log(p_{k})$$

For computational reasons, the simulations of the conformational distributions are usually carried out by assuming that the compound is in a vacuum or within a homogeneous medium. When high-performance computational machines are available, it is possible to exploit more sophisticated methods to consider micro-heterogeneous systems [29].

Conformational Entropy can also be determined experimentally. The first requirement is to pinpoint a variable V that is a function of the conformational distribution for a particular compound. In the case of a homogeneous microscopic system embedding that specific compound, like a dilute solution in a pure solvent, the intermolecular interactions among the conformers can be considered negligible. Therefore, the value of the variable V can be expressed as the weighted sum of the contributions $V_{k}$ of the $N_{C}$ conformers:

(12) $$V=\sum_{k=1}^{N_{C}}v_{k}V_{k}$$

If Equation (12) holds, then the experimentally determined Conformational Entropy $S_{EC}$ will be:

(13) $$S_{EC}=-\sum_{k=1}^{N_{C}}v_{k}log(v_{k})$$

The $v_{k}$ values appearing in Equation (12) often differ from the $p_{k}$ values appearing in Equation (10). The $v_{k}$ values depend not only on the relative abundance of the conformers but also on conformational contribution to the value of the variable V. For instance, if V represents the luminescence intensity, $v_{k}$ will be a function of both the relative amount of the *k*-th conformer and its photoluminescence quantum yield.

In the case of micro-heterogeneous environments, if $N_{E}$ is the number of distinct physicochemical contexts (pcc) wherein each of the $N_{C}$ conformers can reside, then Equation (12) transforms into Equation (14):

(14) $$V=\sum_{k=1}^{N_{C}}\sum_{j=1}^{N_{E}}v_{kj}V_{kj}$$

wherein the subscript $k=1,\ldots ,N_{C}$ represents the number of conformers, whereas the subscript $j=1,\ldots ,N_{E}$ represents the number of distinct micro-environments. In this latter case, the experimentally determined Conformational Entropy $S_{EC}$ will be:

(15) $$S_{EC}=-\sum_{k=1}^{N_{C}}\sum_{j=1}^{N_{E}}v_{kj}log(v_{kj})$$

It is worthwhile noticing that Equations (11) and (13) are relative to the specific case wherein there is just one micro-environment, i.e., $N_{E}=1$.

## 4. Fuzzy Entropy

Any conformational distribution $\bar{\Gamma }$ has the features of a fuzzy set [30,31]. A fuzzy set is a peculiar kind of set that is completely different from a classical Boolean set [32]. For any Boolean set, an element either belong or not to the set. The degree of membership of an element to a Boolean set can be either 1 or 0. On the other hand, for a fuzzy set, an element can belong to it with a degree of membership, which can be any real number included between 0 and 1.

**Figure 2:**
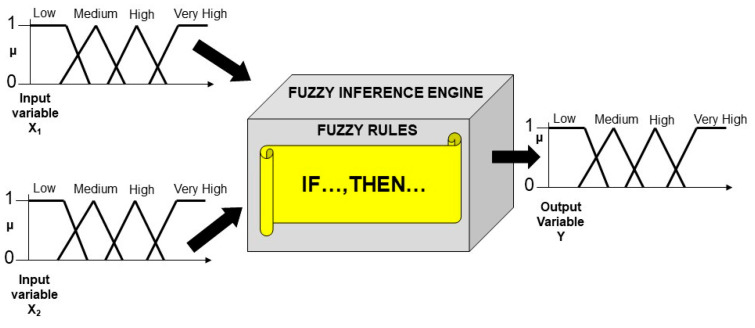
Schematic structure of a hypothetical Fuzzy Logic System based on two-inputs and one-output variables. Each variable has been partitioned into four fuzzy sets. The y-axes represent the degrees of membership (µ) for both the input and output variables. The connection between the input and output variables is guaranteed by the Fuzzy Inference Engine, which is based on fuzzy rules. The fuzzy rules are the IF…, THEN… statements that include the adjectives chosen to label the fuzzy sets.

Boolean sets are the foundations of the crisp binary logic, which allows the manipulation of statements that are either completely true or completely false. On the other hand, fuzzy sets are at the foundations of fuzzy logic. Fuzzy logic represents a good model of human capability to “compute”, i.e., make decisions, using syllogistic statements of the type IF…, THEN…., and words of natural language [33]. Any nonlinear input-output relationship can be modelled by building a Fuzzy Logic System (FLS). The construction of a FLS requires three fundamental steps [34], which are (1) the granulation of the input and output variables, (2) their graduation, and finally (3) the formulation of the fuzzy rules (see Figure 2). The granulation of the variables is operated by partitioning the numerical values of both the input and output variables in fuzzy sets: the number, position, and shape of the fuzzy sets are context-dependent (in Figure 2, each of the two input and one output variables has been partitioned into four fuzzy sets). The graduation of the variables is carried out by labeling each fuzzy sets through an adjective (in Figure 2, the adjectives “Low”, “Medium”, “High”, and “Very High” have been used for labeling the fuzzy sets). Finally, the fuzzy rules are formulated as syllogistic statements of the type IF…, THEN…, wherein the antecedents (i.e., the IF part) will involve the linguistic labels chosen for the input fuzzy sets, whereas the consequent (i.e., the THEN part) will contain the words chosen for labeling the output fuzzy sets. In case of multiple input variables, the antecedents are connected through the AND, OR, NOT operators, which correspond to the operations of intersection, union, and complement of fuzzy sets, respectively. Since the construction and functioning of any FLS rely on a rigorous mathematical procedure that allows the manipulation of not only wholly true and false but also partially true statements, Fuzzy logic has been defined as a rigorous logic of vague reasoning [35].

Any compound that exists as a conformational distribution $\bar{\Gamma }$ can contribute to the granulation of the physicochemical variables, such as temperature, pressure, chemical composition (see Equation (9)), electric and magnetic fields which affect the properties of $\bar{\Gamma }$. The weight coefficients $w_{k}$ (with $k=1,\;2,\ldots ,\;N_{C}$) appearing in the definition of $\bar{\Gamma }$ (Equation (6)) become the degrees of membership of the different $N_{C}$ conformers to the molecular fuzzy set $\bar{\Gamma }$. Its Shannon entropy becomes the fuzzy entropy, $H_{F}$ [36]:

(16) $$H_{F}=-\sum_{k=1}^{N_{C}}w_{k}log(w_{k})$$

When the same compound experiences $N_{E}$ distinct micro-environments, i.e., $N_{E}$ different physicochemical contexts (pcc), then $N_{C}\times N_{E}$ weight coefficients ($w_{kj}$, with $k=1,\ldots ,N_{C}$ and $j=1,\ldots ,N_{E}$) can be defined, and fuzzy entropy becomes:

(17) $$H_{F}=-\sum_{k=1}^{N_{C}}\sum_{j=1}^{N_{E}}w_{kj}log(w_{kj})$$

Molecular conformations play relevant roles in biochemistry as shown for biopolymers in the next paragraph.

## 5. The Biochemical Relevance of Conformations

Proteins are among the most important biopolymers in living cells as also suggested by the etymology of the term “protein”, which derives from the Greek “proteios” meaning “holding the first place” [37]. Over 120 years ago, it was proposed that any protein has just one well-defined three-dimensional (3D) structure determined by its aminoacidic sequence. This idea promoted the formulation of the strongly selective lock-and-key paradigm for the description of protein-and-substrate interplay [38]: one aminoacidic sequence gives rise to one specific 3D structure and one peculiar function. This assumption, also known as Anfinsen’s dogma [39], was supported by data mainly collected through X-ray crystallography.

Data collected through alternative techniques, such as NMR, time-resolved spectroscopies, and high-resolution microscopies, have challenged this old-fashioned paradigm [40]. A new view has emerged: proteins are innately flexible and each of them exists as a collection of many conformers. Such a conformational multiplicity, also called “diversity”, confers multi-functionality to the proteins: every protein exerts several different functions [41]. In other words, conformational diversity gives rise to functional promiscuity [40,42,43]. These two traits render proteins evolvable macromolecules. The structure and function of a protein can evolve depending on the features of the chemical context because the physicochemical properties of the microenvironment affect the conformational distribution. It is now well-established that proteins can moonlight. Moonlighting proteins are proteins in which one polypeptide chain performs more than one physiologically relevant biochemical or biophysical function. So far, hundreds of moonlighting proteins are known [44]. Most of them perform different functions in distinct cellular localizations. Sometimes changes in the cellular concentration of substrates or other ligands can serve as a trigger for changing protein functions. In general, moonlighting proteins undergo structural changes, which can be small movements of surface loops or more drastic modifications of their tertiary or quaternary structures. The latter changes are especially observed in Intrinsically Disordered Proteins (IDPs). It is now recognized that a large portion of the proteome in all domains of life and all viral proteomes examined comprise the so-called IDPs [39,45,46,47,48,49,50], also named as Intrinsically Unfolded Proteins (IUPs), and proteins made up of combinations of structured and Intrinsically Disordered Regions (IDRs) [51]. These proteins are characterized by the lack of a well-defined 3D structure under physiological conditions. They are conformationally heterogeneous. Their conformational heterogeneity enables context-specific functions to emerge in response to environmental conditions and allows a single structural motif to be used in multiple settings [52]. Undoubtedly, the structural flexibility and plasticity represent a functional advantage conferring a wide range of biological functions to IDPs. For instance, they participate in the regulation of cell division, transcription and translation, signal transduction, circadian rhythmicity, phenotypic plasticity, et cetera [39,46,53]. IDPs tend to be multitaskers. They can bind and release multiple targets. They can play as signaling hubs that orchestrate complex cellular events. They also fulfil a relevant role in forming biomolecular condensates [54] that are transient, membrane-less structures that form within cells as reaction chambers.

**Figure 3:**
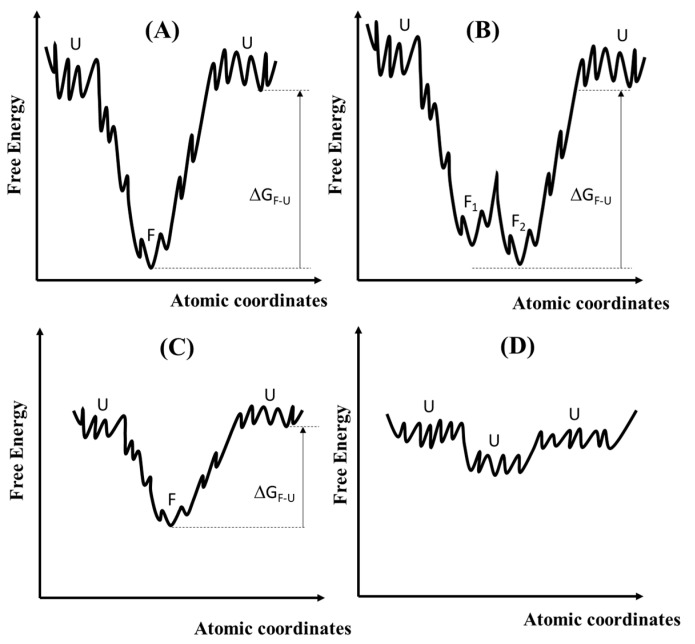
The four graphs present four types of proteins based on the free energies for their conformational structures. The x-axes represent the atomic coordinates involved in the conformational distributions of the proteins. Graph (**A**) represents a Folded Protein (FP) when the folded (F) state has a much lower free energy than the unfolded (U) states. Graph (**B**) refers to a metamorphic protein that has two distinct folded structures (F_1_ and F_2_) with close energies. Graph (**C**) refers to Marginally Stable Proteins (MSPs) for which the free energy difference (${∆G}_{F-U}$) is less than 2 Kcal/mol. Graph (**D**) represents the generic case of an IDP wherein abundant conformers have similar free energies and coexist at ordinary temperatures.

Between the cases of proteins having a well-defined 3D structure, the so-called Folded Proteins (FPs), and the IDPs (or IUPs), there are two other categories, as shown in Figure 3. One is that of metamorphic proteins (graph B in Figure 3) that have two or more folded structures as their native states [55]. They are in thermodynamic equilibrium due to the low activation barrier of refolding [56]. The different structures usually have different functions (Metamorphic proteins that have two or a few more folded structures as their native states can be exploited to process discrete logics). The other category is known as that of Marginally Stable Proteins (MSPs) (see graph C of Figure 3). MSPs exist as an equilibrium mixture of folded and unfolded states [52]. When the folded state (labelled as “F” in Figure 3A) is much more stable than the unfolded states (labelled as “U”), and the free energy difference is larger than 2 Kcal/mol (${∆G}_{F-U}>2\;Kcal/mol\approx 0.09\;eV$) [57], then the protein behaves as a FP (see graph A in Figure 3 where the free energy landscape is depicted as a rugged funnel with the folded state as the most stable conformation or graph B where the free energy landscape has two minima representing the F_1_ and F_2_ states separated by a low barrier). When the free energy difference between the F and U states is less than 2 Kcal/mol, then the protein behaves as an MSP (see graph C in Figure 3). Two or more states (folded and unfolded) coexist in thermodynamic equilibrium. Even small energetic inputs can alter the equilibrium. Therefore, MSPs are powerful sensors both in vitro and in vivo. Finally, graph D represents the case of an IDP or IUP: its free energy landscape is almost flat and rugged with small energetic barriers, permitting mutual interconversion of multiple conformations. IDPs can adopt a continuum of structural states.

IDPs can remain partially disordered even in the bound state in the presence of a partner. This behavior has been named as “fuzziness” [58]. The fuzziness of IDPs may refer to static structural promiscuity, when the IDP has more than one stable bound state, or to dynamic disordered parts of the bound IDP. Fuzziness confers IDPs some functional advantages: interactions with alternative partners and simultaneous interactions with different partners. Due to its intrinsic evolvability, it seems plausible that IDPs may have contributed to the development of early life forms [59,60]. The theories of how life emerged can be grouped into two principal visions [61,62]. One hypothesis highlights the relevance of metabolism, in which small molecules formed an evolving network of reactions driven by an energy source [63]. The other vision contemplates the necessity of a replicator, i.e., a large molecule, such as RNA, formed by chance and capable of replicating [64]. According to the RNA-world hypothesis, life emerged from self-replicating RNA molecules that could catalyze chemical reactions. Isolated RNA is not stably folded. It can be frozen to a stable structure after interacting with a scaffold that might have been made of IDPs. This hypothesis is supported by the evidence that ribosomes are made of RNA interacting with proteins [65].

**Figure 4:**
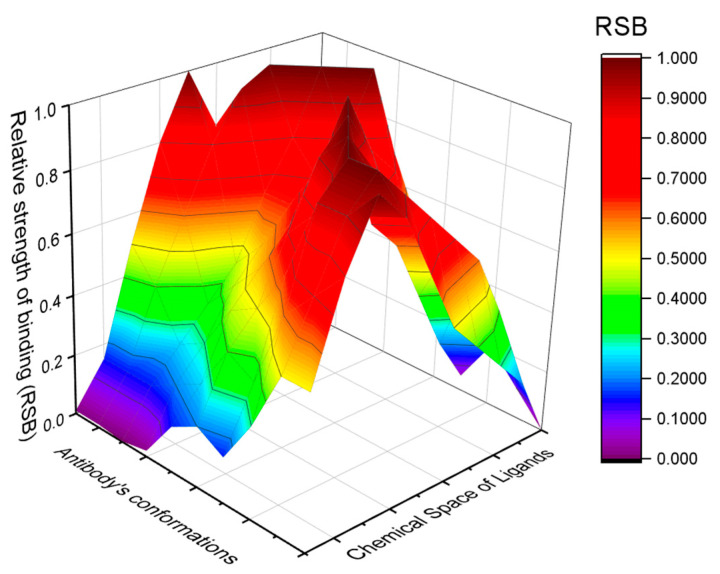
Multi-specificity or cross-reactivity of the immune system’s receptors. Any antibody exists as an ensemble of conformations: each conformer provides a peculiar binding strength towards different ligands. RSB stands for Relative Binding Strength of the antibody’s conformations to the different ligands. The antibody’s conformations and the chemical space of ligands are represented along the binding reaction coordinate.

Protein conformations play relevant roles also in the immune system. A limited repertoire of antibodies and T-cell receptors can recognize and bind to an almost infinite number of antigens [66,67]. This conundrum of “infinite ligands for finite receptors” cannot be solved by the lock-and-key paradigm. The alternative model of pre-existing equilibrium among distinct conformations was envisaged by Pauling in the 1940s [68]. According to this model, an antibody subsists as an ensemble of conformations that coexist in equilibrium, each providing a peculiar binding site and binding specificity (see Figure 4 wherein the Relative Binding Strength of a generic antibody toward the chemical space of ligands is graphed). An antigen binds more strongly to one or a few more conformations of an antibody, thereby biasing the equilibrium towards it or them. This behavior can satisfy the antipodal demands of plasticity in recognition and fidelity during the response [69]. A single antibody can bind to multiple antigens: it cross-reacts. The multiplicity of antibodies’ conformations allows functional diversity without depending on the aminoacidic sequence diversity. The multi-specificity or cross-reactivity (also called promiscuity or degeneracy) of antibodies, T-cell receptors, and other immune system receptors (such as the natural killer cell receptors) confers adaptability to the immune system, but also the pathological capability of turning against the organism is meant to protect [67,70].

Conformations are relevant not only for proteins but also for the two other major biopolymers of living cells, i.e., RNA and DNA.

RNA is a highly flexible and dynamic molecule: it has the intrinsic capability of adopting many interconverting conformations [71]. This ensemble of conformations confers to RNA regulatory roles in many cellular processes, such as transcription, translation, splicing, and nuclear export [72,73,74]. RNA misfolding or aberrant RNA structures caused by mutations or abnormal interactions with other biomolecules can lead to diseases [71].

DNA is a very long polymer that folds hierarchically, through specific proteins (called histones), into several layers of higher-order structures, constituting the genome [75]. The three-dimensional conformational organization of the genome plays an essential role in all the fundamental cellular processes involving DNA, i.e., gene transcription, regulation, and DNA replication [76,77]. Only a small fraction of the genome encodes proteins. Most of the genome exerts regulatory functions. These regulatory functions depend on the three-dimensional conformational organization of the genome. Other striking cellular events strongly depend on the genome’s conformations: they are the physical separation of chromosomes in bacteria, and chromatid demixing and compaction in eukaryotes. Two chromosomes or two chromatids have more conformational degrees of freedom when they are physically separated than when intermingled. The Conformational Entropy of the genome is maximized by chromosome (or chromatid) segregation [78].

## 6. The Logic of Life

The biochemical examples presented in the previous paragraph demonstrate that living cells hinge on molecular conformations. Since conformational distributions are molecular fuzzy sets, it can be inferred that the logic of life is fuzzy, i.e., vague [79,80,81,82]. Although we lack a universally accepted definition of life, the myriad of known life forms shares some features [83,84,85,86,87,88,89,90,91,92].

Firstly, their chemistry: every living organism is made of at least one cell, which is an open system, confined by a membrane, and made of a plethora of interacting chemical compounds, among which the previously mentioned biopolymers, DNA, RNA, and proteins are the basic ingredients.

Secondly, their cycle: every living being starts its existence after its birth from other living matter and ends with its death, becoming inanimate. In between, it develops because it is capable of self-maintaining, self-reproducing [93] and self-protecting against some intruders and harmful elements.

Thirdly, their computing power: every living being exploits matter and energy to encode, collect, store, process, and send information to pursue its goals [94,95]. The basic aims common to every living being are those of surviving and reproducing [96]. Their achievement induces living beings to adapt by adjusting their metabolic processes, to acclimate by turning on and off peculiar genes, and to evolve by changing their genome under an ever-changing environment [97].

The homeostasis and purposefulness of living organisms depend upon a network of regulatory mechanisms, i.e., negative feedback loops [98,99]. Such loops involve proteins, RNA, and DNA molecules. Therefore, these regulatory networks are intrinsically fuzzy. Their fuzziness guarantees adaptability and the capability of making decisions in environments dominated by uncertainty and vagueness, when truth is partial and relative to the context [100]. After all, the most striking successes of fuzzy logic implemented in electronic devices have been achieved in fuzzy controller hardware systems devised to stabilize an inverted pendulum [101], control a space booster rocket and satellite, an automatic aircraft landing system, in pattern recognition, and many other applications which need a swift approximate reasoning [102]. Furthermore, fuzzy logic systems have been built to model and control chaotic systems [103,104,105,106,107,108].

## 7. Mimicking the Logic of Life

The logic of life is fuzzy. The fuzziness of the biochemical circuits derives from the conformational distributions of biopolymers, such as proteins, DNA, and RNA as demonstrated in the previous paragraphs. The multiplicity and diversity of conformers give rise to functional promiscuity: context-specific functions can emerge. Macromolecules and the circuits they generate are evolvable. These features of biochemical networks inspire the research line of Chemical Artificial Intelligence (CAI) [14,15,16,109]. In CAI, the parallelism of chemical reactions is exploited [110,111,112]. The inputs and outputs are physicochemical variables. Particularly valuable is light that bridges the macroscopic and molecular worlds [113,114,115].

The purpose of CAI is to exploit molecular, supramolecular, and systems chemistry to design and implement chemical systems in wetware (i.e., in fluid solutions) to mimic some performances of human and biochemical intelligence. The ultimate and ambitious goal of CAI is the development of chemical robots [16,116,117,118]. Chemical robots are supposed to be autonomous molecular assemblies, confined within a membrane, having micrometric dimensions and being provided with four modules: (1) a sensory, (2) a neural network-type, (3) an effector, and (4) a metabolic module, respectively. The sensory module collects physicochemical data about the surrounding micro-environment and the internal state of the robot. The neural network-type module processes the sensory data and make decisions, triggering the action of the effector module that can act upon the embedding environment. The intelligent activities of the chemical robots must be energetically sustained by the metabolic module. Unicellular micro-organisms are prototypes of chemical robots. Therefore, the implementation of chemical robots can reside within the broad realm of synthetic biology. More specifically, chemical robots are supposed to be fabricated through the bottom-up approach by assembling all the necessary modules required to ensure the expected performances, as if they were synthetic (or artificial) cells [119,120,121]. Chemical robots will be capable of processing fuzzy logic if their modules will rely on molecular fuzzy sets [30,31], which are conformational distributions, especially those associated with macromolecules. Mixing conformational distributions belonging to distinct compounds, which are sensitive to the same kind of physicochemical variables, allows the granulation and graduation of the physicochemical variables, in analogy to the human sensory system [122,123,124]. When the molecular fuzzy sets are nodes of network-type chemical circuits, they allow the implementation of chemical fuzzy neural networks [125]. If these networks are recurrent because they include feedback actions, they become the chemical implementation of neuro-fuzzy algorithms, which allow to adapt, learn and make swift and reasonable decisions in uncertain and vague conditions. The cognitive functions of the chemical fuzzy neural networks can be increased by assembling distinct chemical robots and generating a sort of swarm or collective intelligence [126,127]. Such webs or swarm of chemical robots might establish chemical links with unicellular organisms or more in general living cells, and originate the so-called Internet of Bio-Nano Things (IoBNTs) [128].

Chemical robots and the hybrid IoBNTs, capable of processing fuzzy logic, promise to colonize the microscopic world and be helpful in many fields, such as in diagnosis and therapies for human health, in safeguarding and cleaning natural ecosystems and urban areas [16,129,130,131].

## 8. Conclusions

Fuzzy logic is not only a good model of human capability to compute (i.e., make decisions) with words [33,123,125], but based on the results presented in this work, it is also appropriate for interpreting decision making even at the level of single cells. The conformational distributions’ fuzziness allows the implementation of functional promiscuity and adaptability at the molecular level. When conformational distributions of distinct compounds are organized in networks, they give rise to higher cognitive functions, such as the capability of making reasonable decisions in environments dominated by uncertainty and vagueness. This awareness will be exploited in the design and implementation of chemical artificial intelligent systems and chemical robots that will help humans to colonize the molecular world [132] and fight against diseases in living beings and pollution in ecosystems.

## Data Availability

No new data were created or analyzed in this study. Data sharing is not applicable to this study.

## References

[1] Whitlock H.W. (1998). On the structure of total synthesis of complex natural products. J. Org. Chem..

[2] Barone R., Chanon M. (2001). A new simple approach to chemical complexity. Application to the synthesis of natural products. J. Chem. Inf. Comput. Sci..

[3] Bertz S.H. (2003). Complexity of synthetic reactions. The use of complexity indices to evaluate reactions, transforms and disconnections. New J. Chem..

[4] Allu T.K., Oprea T.I. (2005). Rapid evaluation of synthetic and molecular complexity for in silico chemistry. J. Chem. Inf. Model..

[5] Ertl P., Schuffenhauer A. (2009). Estimation of synthetic accessibility score of drug-like molecules based on molecular complexity and fragment contributions. J. Cheminf..

[6] Leach A.R., Hann M.M. (2011). Molecular complexity and fragment-based drug discovery: Ten years on. Curr. Opin. Chem. Biol..

[7] Bonnet P. (2012). Is chemical synthetic accessibility computationally predictable for drug and lead-like molecules? A comparative assessment between medicinal and computational chemists. Eur. J. Med. Chem..

[8] Méndez-Lucio O., Medina-Franco J.L. (2017). The many roles of molecular complexity in drug discovery. Drug Discov. Today.

[9] Rashevsky N. (1955). Life, information theory, and topology. Bull. Math. Biophys..

[10] Schuffenhauer A., Brown N., Selzer P., Ertl P., Jacoby E. (2006). Relationship between molecular complexity, biological activity, and structural diversity. J. Chem. Inf. Model..

[11] Marshall S.M., Mathis C., Carrick E., Keenan G., Cooper G.J., Graham H., Craven M., Gromski P.S., Moore D.G., Walker S.I. (2021). Identifying molecules as biosignatures with assembly theory and mass spectrometry. Nat. Commun..

[12] d’Hendecourt L.L. (2011). Molecular complexity in astrophysical environments: From astrochemistry to “astrobiology”?. EPJ Web Conf..

[13] García-Sánchez M., Jiménez-Serra I., Puente-Sánchez F., Aguirre J. (2022). The emergence of interstellar molecular complexity explained by interacting networks. Proc. Natl. Acad. Sci. USA.

[14] Gentili P.L. (2013). Small steps towards the development of chemical artificial intelligent systems. RSC Adv..

[15] Gentili P.L., Stano P. (2023). Tracing a new path in the field of AI and robotics: Mimicking human intelligence through chemistry. Part I: Molecular and supramolecular chemistry. Front. Robot. AI.

[16] Gentili P.L., Stano P. (2023). Tracing a new path in the field of AI and robotics: Mimicking human intelligence through chemistry. Part II: Systems chemistry. Front. Robot. AI.

[17] des Cloizeaux J., Jannink G. (1990). Polymers in Solution: Their Modeling and Structure.

[18] de Gennes P.G. (1990). Introduction to Polymer Dynamics.

[19] Zuckerman D.M. (2010). Statistical Physics of Biomolecules: An Introduction.

[20] Bonchev D.G., Rouvray D.H. (2003). Complexity: Introduction and Fundamentals.

[21] Sabirov D.S., Shepelevich I.S. (2021). Information Entropy in Chemistry: An Overview. Entropy.

[22] Geerlings P., Borgooa A. (2011). Information carriers and (reading them through) information theory in quantum chemistry. Phys. Chem. Chem. Phys..

[23] Selzer P., Roth H.-J., Ertl P., Schuffenhauer A. (2005). Complex molecules: Do they add value?. Curr. Opin. Chem. Biol..

[24] Sheridan R.P., Zorn N., Sherer E.C., Campeau L.-C., Chang C.Z., Cumming J., Maddess M.L., Nantermet P.G., Sinz C.G., O’Shea P.D. (2014). Modeling a Crowdsourced Definition of Molecular Complexity. J. Chem. Inf. Model..

[25] von Korff M., Sander T. (2019). Molecular Complexity Calculated by Fractal Dimension. Sci. Rep..

[26] Feldman D.P. (2012). Chaos and Fractals: An Elementary Introduction.

[27] Chiolerio A., Vitiello G., Dehshibi M.M., Adamatzky A. (2023). Living Plants Ecosystem Sensing: A Quantum Bridge between Thermodynamics and Bioelectricity. Biomimetics.

[28] Trinajstic N. (1992). Chemical Graph Theory.

[29] Chung L.W., Sameera W.M.C., Ramozzi R., Page A.J., Hatanaka M., Petrova G.P., Harris T.V., Li X., Ke Z., Liu F. (2015). The ONIOM method and its applications. Chem. Rev..

[30] Gentili P.L. (2014). The fuzziness of a chromogenic spirooxazine. Dye. Pigment..

[31] Gentili P.L. (2018). The fuzziness of the molecular world and its perspectives. Molecules.

[32] Zadeh L.A. (1965). Fuzzy sets. Inf. Control.

[33] Zadeh L.A. (1999). From computing with numbers to computing with words. From manipulation of measurements to manipulation of perceptions. IEEE Trans. Circuits Syst. I Fundam. Theory Appl..

[34] Mendel J.M. (1995). Fuzzy logic systems for engineering: A tutorial. Proc. IEEE.

[35] Zadeh L.A. (1997). Toward a theory of fuzzy information granulation and its centrality in human reasoning and fuzzy logic. Fuzzy Sets Syst..

[36] Gentili P.L., Perez-Mercader J. (2022). Quantitative estimation of chemical microheterogeneity through the determination of fuzzy entropy. Front. Chem..

[37] Vickery H.B. (1950). The origin of the word protein. Yale J. Biol. Med..

[38] Lemieux R.U., Spohr U. (1994). How Emil Fischer was led to the lock and key concept for enzyme specificity. Adv. Carbohydr. Chem. Biochem..

[39] Kulkarni P., Leite V.B., Roy S., Bhattacharyya S., Mohanty A., Achuthan S., Singh D., Appadurai R., Rangarajan G., Weninger K. (2022). Intrinsically disordered proteins: Ensembles at the limits of Anfinsen’s dogma. Biophys. Rev..

[40] James L.C., Tawfik D.S. (2003). Conformational diversity and protein evolution–a 60-year-old hypothesis revisited. Trends Biochem. Sci..

[41] Ma B., Shatsky M., Wolfson H.J., Nussinov R. (2002). Multiple diverse ligands binding at a single protein site: A matter of pre-existing populations. Protein Sci..

[42] Vavouri T., Semple J.I., Garcia-Verdugo R., Lehner B. (2009). Intrinsic protein disorder and interaction promiscuity are widely associated with dosage sensitivity. Cell.

[43] Marcotte E.M., Tsechansky M. (2009). Disorder, promiscuity, and toxic partnerships. Cell.

[44] Liu H., Jeffery C.J. (2020). Moonlighting Proteins in the Fuzzy Logic of Cellular Metabolism. Molecules.

[45] Wright P.E., Dyson H.J. (1999). Intrinsically unstructured proteins: Re-assessing the protein structure-function paradigm. J. Mol. Biol..

[46] Jakobs U., Kriwacki R., Uversky V.N. (2014). Conditionally and Transiently Disordered Proteins: Awakening Cryptic Disorder to Regulate Protein Function. Chem. Rev..

[47] Vucetic S., Brown C.J., Dunker A.K., Obradovic Z. (2003). Flavors of protein disorder. Proteins Struct. Funct. Genet..

[48] Ward J., Sodhi J., McGuffin L., Buxton B., Jones D. (2004). Prediction and Functional Analysis of Native Disorder in Proteins from the Three Kingdoms of Life. J. Mol. Biol..

[49] Peng Z., Yan J., Fan X., Mizianty M.J., Xue B., Wang K., Hu G., Uversky V.N., Kurgan L. (2015). Exceptionally abundant exceptions: Comprehensive characterization of intrinsic disorder in all domains of life. Cell. Mol. Life Sci..

[50] Piovesan D., Tabaro F., Mičetić I., Necci M., Quaglia F., Oldfield C.J., Aspromonte M.C., Davey N.E., Davidović R., Dosztányi Z. (2017). DisProt 7.0: A major update of the database of disordered proteins. Nucleic Acids Res..

[51] Van Der Lee R., Buljan M., Lang B., Weatheritt R.J., Daughdrill G.W., Dunker A.K., Fuxreiter M., Gough J., Gsponer J., Jones D.T. (2014). Classification of intrinsically disordered regions and proteins. Chem. Rev..

[52] Pricer R., Gestwicki J.E., Mapp A.K. (2017). From Fuzzy to Function: The New Frontier of Protein-Protein Interactions. Acc. Chem. Res..

[53] Kulkarni P. (2020). Intrinsically Disordered Proteins: Insights from Poincaré, Waddington, and Lamarck. Biomolecules.

[54] Holehouse A.S., Salvi N. (2019). Chapter 7—IDPs and IDRs in biomolecular condensates. Intrinsically Disordered Proteins.

[55] Lella M., Mahalakshmi R. (2017). Metamorphic Proteins: Emergence of Dual Protein Folds from One Primary Sequence. Biochemistry.

[56] Goodchild S.C., Curmi P.M.G., Brown L.J. (2011). Structural gymnastics of multifunctional metamorphic proteins. Biophys. Rev..

[57] Pastore A., Martin S.R., Temussi P.A. (2019). Generalized View of Protein Folding: In Medio Stat Virtus. J. Am. Chem. Soc..

[58] Tompa P., Fuxreiter M. (2008). Fuzzy complexes: Polymorphism and structural disorder in protein–protein interactions. Trends Biochem. Sci..

[59] Kulkarni P., Uversky V.N. (2018). Intrinsically Disordered Proteins and the Janus Challenge. Biomolecules.

[60] Kulkarni P., Uversky V.N. (2018). Intrinsically Disordered Proteins: The Dark Horse of the Dark Proteome. Proteomics.

[61] Shapiro R. (2007). A simpler origin for life. Sci. Am..

[62] Ruiz-Mirazo K., Briones C., de la Escosura A. (2014). Prebiotic systems chemistry: New perspectives for the origins of life. Chem. Rev..

[63] Shapiro R. (2006). Small molecule interactions were central to the origin of life. R. Q. Rev. Biol..

[64] Higgs P.G., Lehman N. (2015). The RNA World: Molecular cooperation at the origins of life. Nat. Rev. Genet..

[65] Katsnelson A. (2020). Did disordered proteins help launch life on Earth?. ACS Cent. Sci..

[66] Keskin O. (2007). Binding induced conformational changes of proteins correlate with their intrinsic fluctuations: A case study of antibodies. BMC Struct. Biol..

[67] Mariuzza R.A. (2006). Multiple Paths to Multispecificity. Immunity.

[68] Pauling L. (1940). A theory of the structure and process of formation of antibodies. J. Am. Chem. Soc..

[69] Manivel V., Sahoo N.C., Salunke D.M., Rao K.V. (2000). Maturation of an antibody response is governed by modulations in flexibility of the antigen-combining site. Immunity.

[70] Oldstone M.B. (1998). Molecular mimicry and immune-mediated diseases. FASEB J..

[71] Payal G., Khadake R.M., Panja S., Shinde K., Rode A.B. (2022). Alternative RNA Conformations: Companion or Combatant. Genes.

[72] Cross S.T., Michalski D., Miller M.R., Wilusz J. (2019). RNA regulatory processes in RNA virus biology. Wiley Interdiscip. Rev. RNA.

[73] Masse E., Majdalani N., Gottesman S. (2003). Regulatory roles for small RNAs in bacteria. Curr. Opin. Microbiol..

[74] Stepanov G.A., Filippova J.A., Komissarov A.B., Kuligina E.V., Richter V.A., Semenov D.V. (2015). Regulatory role of small nucleolar RNAs in human diseases. BioMed Res. Int..

[75] Woodcock C.L. (2006). Chromatin architecture. Curr. Opin. Struct. Biol..

[76] Lin X., Qi Y., Latham A.P., Zhang B. (2021). Multiscale modeling of genome organization with maximum entropy optimization. J. Chem. Phys..

[77] Cremer T., Cremer M., Hübner B., Strickfaden H., Smeets D., Popken J., Sterr M., Markaki Y., Rippe K., Cremer C. (2015). The 4D nucleome: Evidence for a dynamic nuclear landscape based on co-aligned active and inactive nuclear compartments. FEBS Lett..

[78] Jun S., Wright A. (2010). Entropy as the driver of chromosome segregation. Nat. Rev. Microbiol..

[79] Gentili P.L., Syropoulos A., Syropoulos A., Papadopoulos B.K. (2021). 6 Vagueness in chemistry. Vagueness in the Exact Sciences: Impacts in Mathematics, Physics, Chemistry, Biology, Medicine, Engineering and Computing.

[80] Peretó J. (2012). Out of fuzzy chemistry: From prebiotic chemistry to metabolic networks. Chem. Soc. Rev..

[81] Gilles B., Bartik K., Reisse J. (2010). Is it useful to have a clear-cut definition of life? On the use of fuzzy logic in prebiotic chemistry. Orig. Life Evol. Biosph..

[82] Gilles B., Bartik K., Reisse J. (2011). Prebiotic chemistry: A fuzzy field. Comptes Rendus Chim..

[83] Cleland C., Chyba C. (2007). Does life have a definition?. Planets and Life: The Emerging Science of Astrobiology.

[84] Cornish-Bowden A., Cárdenas M.L. (2020). Contrasting theories of life: Historical context, current theories. In search of an ideal theory. Biosystems.

[85] Mariscal C., Doolittle W.F. (2020). Life and life only: A radical alternative to life definitionism. Synthese.

[86] Ruiz-Mirazo K., Peretó J., Moreno A. (2004). A universal definition of life: Autonomy and open-ended evolution. Orig. Life Evol. Biosph..

[87] Vitas M., Dobovišek A. (2019). Toward a general definition of life. Orig. Life Evol. Biosph..

[88] Witzany G. (2020). What is life?. Front. Astron. Space Sci..

[89] Bartlett S., Wong M.L. (2020). Defining lyfe in the universe: From three privileged functions to four pillars. Life.

[90] Gentili P.L. (2021). Why is Complexity Science valuable for reaching the goals of the UN 2030 Agenda?. Rend. Fis. Acc. Lincei.

[91] Muñuzuri A.P., Pérez-Mercader J. (2022). Unified representation of Life’s basic properties by a 3-species Stochastic Cubic Autocatalytic Reaction-Diffusion system of equations. Phys. Life Rev..

[92] Gentili P.L. (2023). The Relevant Role that Natural Computing Can Play in the Development of Complexity Science. Int. J. Unconv. Comput..

[93] Maturana H.R., Valera F.J., Cohen R.S., Wartofsky M.V. (1980). Autopoiesis and cognition: The realization of the living. Boston Studies in the Philosophy of Science.

[94] Walker S.I., Davies P.C.W., Ellis G.F.R. (2017). From Matter to Life.

[95] Roederer J. (2005). Information and Its Role in Nature.

[96] Monod J. (1971). Chance and Necessity: Essay on the Natural Philosophy of Modern Biology.

[97] Rojdestvenski I., Cottam M.G., Oquist G., Huner N. (2003). Thermodynamics of complexity. Phys. A.

[98] Korzeniewski B.J. (2001). Cybernetic Formulation of the Definition of Life. Theor. Biol..

[99] Bartlett S., Louapre D. (2022). Provenance of life: Chemical autonomous agents surviving through associative learning. Phys. Rev. E.

[100] Dubois D., Prade H. (2001). Possibility theory, probability theory and multiple-valued logics: A clarification. Ann. Math. Artif. Intell..

[101] Yamakawa T. (1989). Stabilization of an inverted pendulum by a high-speed fuzzy logic controller hardware system. Fuzzy Sets Syst..

[102] Kandel A., Langholz G. (1998). Fuzzy Hardware: Architectures and Applications.

[103] Calvo O., Cartwright J.H. (1998). Fuzzy control of chaos. Int. J. Bifurc. Chaos.

[104] Precup R.-E., Tomescu M.L. (2015). Stable fuzzy logic control of a general class of chaotic systems. Neural Comput. Appl..

[105] Gentili P.L., Giubila M.S., Heron B.M. (2017). Processing Binary and Fuzzy Logic by Chaotic Time Series Generated by a Hydrodynamic Photochemical Oscillator. ChemPhysChem.

[106] Hayashi K., Gotoda H., Gentili P.L. (2016). Probing and exploiting the chaotic dynamics of a hydrodynamic photochemical oscillator to implement all the basic binary logic functions. Chaos.

[107] Gentili P.L., Gotoda H., Dolnik M., Epstein I.R. (2015). Analysis and prediction of aperiodic hydrodynamic oscillatory time series by feedforward neural networks, fuzzy logic, and a local nonlinear predictor. Chaos.

[108] Gentili P.L. (2018). Untangling Complex Systems: A Grand Challenge for Science.

[109] Gentili P.L., Szaciłowski K., Adamatzky A. (2023). Editorial: Approaching human intelligence through chemical systems: Development of unconventional chemical artificial intelligence. Front. Chem..

[110] Nagahara H., Ichino T., Yoshikawa K. (2004). Direction detector on an excitable field: Field computation with coincidence detection. Phys. Rev. E.

[111] Górecki J., Górecka J.N., Yoshikawa K., Igarashi Y., Nagahara H. (2005). Sensing the distance to a source of periodic oscillations in a nonlinear chemical medium with the output information coded in frequency of excitation pulses. Phys. Rev. E.

[112] Tomassoli L., Silva-Dias L., Dolnik M., Epstein I.R., Germani R., Gentili P.L. (2024). Neuromorphic Engineering in Wetware: Discriminating Acoustic Frequencies through Their Effects on Chemical Waves. J. Phys. Chem. B.

[113] Gentili P.L., Micheau J.-C. (2020). Light and chemical oscillations: Review and perspectives. J. Photochem. Photobiol. C Photochem. Rev..

[114] Gentili P.L. (2011). Molecular Processors: From Qubits to Fuzzy Logic. ChemPhysChem.

[115] Gentili P.L. (2022). Photochromic and Luminescent materials for the development of Chemical Artificial Intelligence. Dye. Pigment..

[116] Hagiya M., Konagaya A., Kobayashi S., Saito H., Murata S. (2014). Molecular robots with sensors and intelligence. Acc. Chem. Res..

[117] Murata S., Toyota T., Nomura S.I.M., Nakakuki T., Kuzuya A. (2022). Molecular Cybernetics: Challenges toward cellular chemical artificial intelligence. Adv. Funct. Mat..

[118] Chiolerio A., Quadrelli M.B. (2017). Smart Fluid Systems: The Advent of Autonomous Liquid Robotics. Adv. Sci..

[119] Guindani C., Silva L.C., Cao S., Ivanov T., Landfester K. (2022). Synthetic cells: From simple bio-inspired modules to sophisticated integrated systems. Angew. Chem. Int. Ed..

[120] Luisi P.L. (2002). Toward the engineering of minimal living cells. Anat. Rec..

[121] Gentili P.L., Stano P. (2023). Monitoring the advancements in the technology of artificial cells by determining their complexity degree: Hints from complex systems descriptors. Front. Bioeng. Biotechnol..

[122] Gentili P.L., Rightler A.L., Heron B.M., Gabbutt C.D. (2016). Extending human perception of electromagnetic radiation to the UV region through biologically inspired photochromic fuzzy logic (BIPFUL) systems. Chem. Commun..

[123] Gentili P.L. (2014). The human sensory system as a collection of specialized fuzzifiers: A conceptual framework to inspire new artificial intelligent systems computing with words. J. Intell. Fuzzy Syst..

[124] Gentili P.L. (2021). Establishing a New Link between Fuzzy Logic, Neuroscience, and Quantum Mechanics through Bayesian Probability: Perspectives in Artificial Intelligence and Unconventional Computing. Molecules.

[125] Gentili P.L., Stano P. (2022). Chemical neural networks inside synthetic cells? A proposal for their realization and modeling. Front. Bioeng. Biotechnol..

[126] Couzin I. (2007). Collective minds. Nature.

[127] Watson R., Levin M. (2023). The collective intelligence of evolution and development. Collect. Intell..

[128] Stano P., Gentili P.L., Damiano L., Magarini M. (2023). A role for bottom-up synthetic cells in the internet of bio-nano things?. Molecules.

[129] Tran H.H., Watkins A., Oh M.J., Babeer A., Schaer T.P., Steager E., Koo H. (2023). Targeting biofilm infections in humans using small scale robotics. Trends Biotechnol..

[130] Shapiro E. (2012). A mechanical Turing machine: Blueprint for a biomolecular computer. Interface Focus.

[131] Kuscu M., Unluturk B.D. (2021). Internet of bio-nano things: A review of applications, enabling technologies and key challenges. ITU J. Future Evol. Technol..

[132] Kurzweil R. (2014). The Singularity Is Near.

